# The genetic cause of neurodevelopmental disorders in 30 consanguineous families

**DOI:** 10.3389/fmed.2024.1424753

**Published:** 2024-08-30

**Authors:** Sohail Aziz Paracha, Shoaib Nawaz, Muhammad Tahir Sarwar, Asmat Shaheen, Gohar Zaman, Jawad Ahmed, Fahim Shah, Sundus Khwaja, Abid Jan, Nida Khan, Mohammad Azhar Kamal, Qamre Alam, Safdar Abbas, Saman Farman, Ahmed Waqas, Afnan Alkathiri, Abdullah Hamadi, Federico Santoni, Naseeb Ullah, Bisma Khalid, Stylianos E. Antonarakis, Khalid A Fakhro, Muhammad Umair, Muhammad Ansar

**Affiliations:** ^1^Department of Anatomy, Institute of Medical Sciences (KIMS), Khyber Medical University, Kohat, Pakistan; ^2^Department of Human Genetics, Sidra Medicine, Doha, Qatar; ^3^Department of Molecular Biology and Genetics, Khyber Medical University, Institute of Basic Sciences, Peshawar, Pakistan; ^4^Department of Biochemistry, Institute of Medical Sciences (KIMS), Khyber Medical University, Kohat, Pakistan; ^5^Department of Computer Science, Abbottabad University of Science and Technology, Havelin, Pakistan; ^6^Department of Microbiology, Khyber Medical University, Institute of Basic Sciences, Peshawar, Pakistan; ^7^Department of Medicine, District Headquarter Hospital, K.D.A, Kohat, Pakistan; ^8^Department of Biotechnology, University of Azad Jammu and Kashmir, Muzaffarabad, Pakistan; ^9^Department of Biotechnology and Genetic Engineering, Kohat University of Science and Technology, Kohat, Pakistan; ^10^Department of Obstetrics and Gynecology, Hayatabad Medical Complex, Peshawar, Pakistan; ^11^Department of Pharmaceutics, College of Pharmacy, Prince Sattam Bin Abdulaziz University, Alkharj, Saudi Arabia; ^12^Molecular Genomics and Precision Medicine, Express Med Diagnostics and Research, Manama, Bahrain; ^13^Department of Biological Science, Dartmouth College, Hanover, NH, United States; ^14^Faculty of Biosciences, COMSATS University, Islamabad, Pakistan; ^15^Department of Zoology, Emerson University, Multan, Pakistan; ^16^Medical Genetics, Laboratory Medicine Department, Faculty of Applied Medical Sciences, Albaha University, Albaha, Saudi Arabia; ^17^Faculty of Applied Medical Sciences, Department of Medical Laboratory Technology, University of Tabuk, Tabuk, Saudi Arabia; ^18^Service of Endocrinology, Diabetes, and Metabolism, University Hospital of Lausanne, Lausanne, Switzerland; ^19^Faculty of Biology and Medicine, University of Lausanne, Lausanne, Switzerland; ^20^National Research Council (CNR), Rome, Italy; ^21^Institute of Biochemistry, University of Balochistan, Quetta, Pakistan; ^22^Department of Genetic Medicine and Development, School of Medicine, University of Geneva, Geneva, Switzerland; ^23^Department of Genetic Medicine, Weill Cornell Medical College, Doha, Qatar; ^24^College of Health and Life Sciences, Hamad Bin Khalifa University, Doha, Qatar; ^25^King Abdullah International Medical Research Center (KAIMRC), Department of Medical Genomics Research, King Saud Bin Abdulaziz University for Health Sciences, Ministry of National Guard Health Affairs (MNGH), Riyadh, Saudi Arabia; ^26^Department of Life Sciences, School of Science, University of Management and Technology (UMT), Lahore, Pakistan; ^27^Department of Ophthalmology, University of Lausanne, Jules Gonin Eye Hospital, Fondation Asile Des Aveugles, Lausanne, Switzerland; ^28^Advanced Molecular Genetics and Genomics Disease Research and Treatment Centre, Dow University of Health Sciences, Karachi, Pakistan

**Keywords:** neurodevelopmental disorders, 30 families, ASPM, novel variants, WES, consanguineous marriages

## Abstract

**Objective:**

This study aims to clinically and genetically assess 30 unrelated consanguineous Pakistani families from various ethnic backgrounds, all exhibiting features of neurodevelopmental disorders (NDDs).

**Methods:**

We conducted clinical, genetic, biochemical, and molecular analyses on 30 consanguineous families with NDDs enrolled from various regions of Pakistan. The likely molecular causes of primary microcephaly and NDDs were identified. Detailed clinical investigations and molecular diagnoses were performed using whole exome sequencing (WES) of the proband, followed by Sanger sequencing for validation and segregation in the available family members of the affected families.

**Results:**

WES identified likely disease-causing homozygous variants in 30 unrelated consanguineous families. Six families presented newly described variants in known NDD-related genes: *ABAT* (c.1439 T > G; p.Phe480Cys) [OMIM613163], *SLC12A6* (c.2865_2865insT; p.Glu955Asnfs*5) [OMIM 218000], *SHANK3* (c.1305-3_1,305-2delTT; p.Gln29-_Gly305del) [OMIM 606232], *BCKDK* (c.356_356insC; p.Gly119Alafs*24) [OMIM 614923], *DDHD2* (c.2065G > T; p.Asp689Tyr) [OMIM 615033], *ERCC2* (c.1255G > A; p.Glu419Lys) [OMIM 610756]. Additionally, 12 families had previously reported disease-causing variants associated with different types of NDDs: *ATRX* (c.109C > T; p.Arg37*) [OMIM 309580], *GPR56* [*ADGRG1*] (c.1423C > T; p.Arg475*) [OMIM 606854], *NAGLU* (c.1694G > A; p.Arg565Gln) [OMIM 252920], *DOLK* (c.3G > A; p.Met1Ile) [OMIM 610768], *GPT2* (c.815C > T; p.Ser272Leu) [OMIM 616281], *DYNC1I2* (c.607 + 1G > A; p.?) [OMIM 618492], *FBXL3* (c.885delT; p.Leu295Phefs25*) [OMIM 606220], *LINGO1* (c.869G > A; p.Arg290His) [OMIM 618103], and *ASPM* (c.3978G > A; Trp1326*, c.9557C > G; p.Ser3186*, c.6994C > T; p.Arg2332*) [OMIM 608716]. All the identified variants showed segregation compatible with autosomal recessive inheritance.

**Conclusion:**

In the present study, we observed a high frequency of *ASPM* variants in the genetic analysis of 30 consanguineous families exhibiting features of NDDs, particularly those associated with autosomal recessive primary microcephaly. These findings contribute to studies on genotype–phenotype correlation, genetic counseling for families, and a deeper understanding of human brain function and development.

## 1 Introduction

Neurodevelopmental disorders (NDDs) are highly heterogeneous and characterized by diverse phenotypic presentations such as cognition impairment and defective behavioral and psychomotor developmental skills. The global prevalence of these disorders has been estimated to be >3% (1). Moreover, the prevalence of NDD was estimated to vary between 1 and 3% worldwide. However, different countries and ethnicities have different prevalences depending on the population history and consanguinity practices (2). For children aged 2 to 6 years, NDD prevalence ranges from 2.9 to 18.7%, while for those aged 6 to 9 years, it ranges from 6.5 to 18.5% (3). Additional features commonly observed in NDD patients include hearing impairment, speech and language disorders, intellectual disability (ID), epilepsy, and learning disorders (4). The phenotypic spectrum of NDDs encompasses intellectual disability (ID), communication disorders, autism spectrum disorder (ASD), attention-deficit/hyperactivity disorder (ADHD), and several other neurodevelopmental motor disorders (5)..

Several genetic and environmental factors have been reported as etiological factors of NDDs (6). However, the main causal factor for these disorders is pathogenic genetic variability, the recognition of which is key for precise (early) diagnosis and proper genetic counseling (7). These genetic aberrations mainly occur due to consanguineous marriages (for autosomal recessive phenotypes) or *de novo* autosomal dominant variants in the offspring of unaffected parents (8). The knowledge-based database OMIM contains >1,000 genes associated with NDD. However, the full spectrum of genes related to NDD is unknown and may include thousands of additional genes (9). To date, more than 1,500 genes have been reported to be responsible for NDDs (10). Several large-scale genomic studies have facilitated the identification of *de novo* mutations in protein-coding genes (11, 12). One of the recent studies from the Wellcome Sanger Institute identified 285 genes significantly associated with developmental disorders and suggested that more than 1,000 such genes may remain undiscovered (13).

The consanguinity rate in Pakistan is approaching 70%, leading to a higher prevalence of recessive genetic disorders such as NDDs. Studying consanguineous families provides a unique opportunity to identify pathogenic variants in the genes responsible for these disorders, as these families harbor extensive regions of homozygosity in their genomes. Whole exome sequencing (WES) has proven to be a powerful and cost-effective methodology for molecular diagnosis and discovering disease-causing genes in families with suspected autosomal recessive phenotypes.

In the current study, we describe 30 unrelated consanguineous Pakistani families from various ethnic backgrounds that exhibit features of NDDs. In addition, WES revealed novel and previously reported variants in several genes associated with different types of NDDs.

## 2 Materials and methods

### 2.1 Patients and study approval

All the families with NDDs were recruited from various remote regions of Pakistan, including the provinces of Khyber Pakhtunkhwa (KPK) and Punjab. The studies involving humans were approved by the University Hospitals of Geneva (Protocol CER 11–036), the Institutional Review Board (IRB) of Khyber Medical University, Peshawar, Pakistan, and the Department of Life Sciences, School of Science, University of Management and Technology (UMT), Lahore, Pakistan. The studies were conducted in accordance with local legislation and institutional requirements. Written informed consent for participation in this study was provided by the participants’ legal guardians/next of kin. Additionally, written informed consent was obtained from the individual(s) to publish any potentially identifiable images or data in this article. Clinical evaluations were performed at local government hospitals. Whole exome sequencing, data analysis, and Sanger sequencing for several families were performed at respective institutes in Saudi Arabia and Qatar.

### 2.2 Genomic DNA isolation

**Figure 1:**
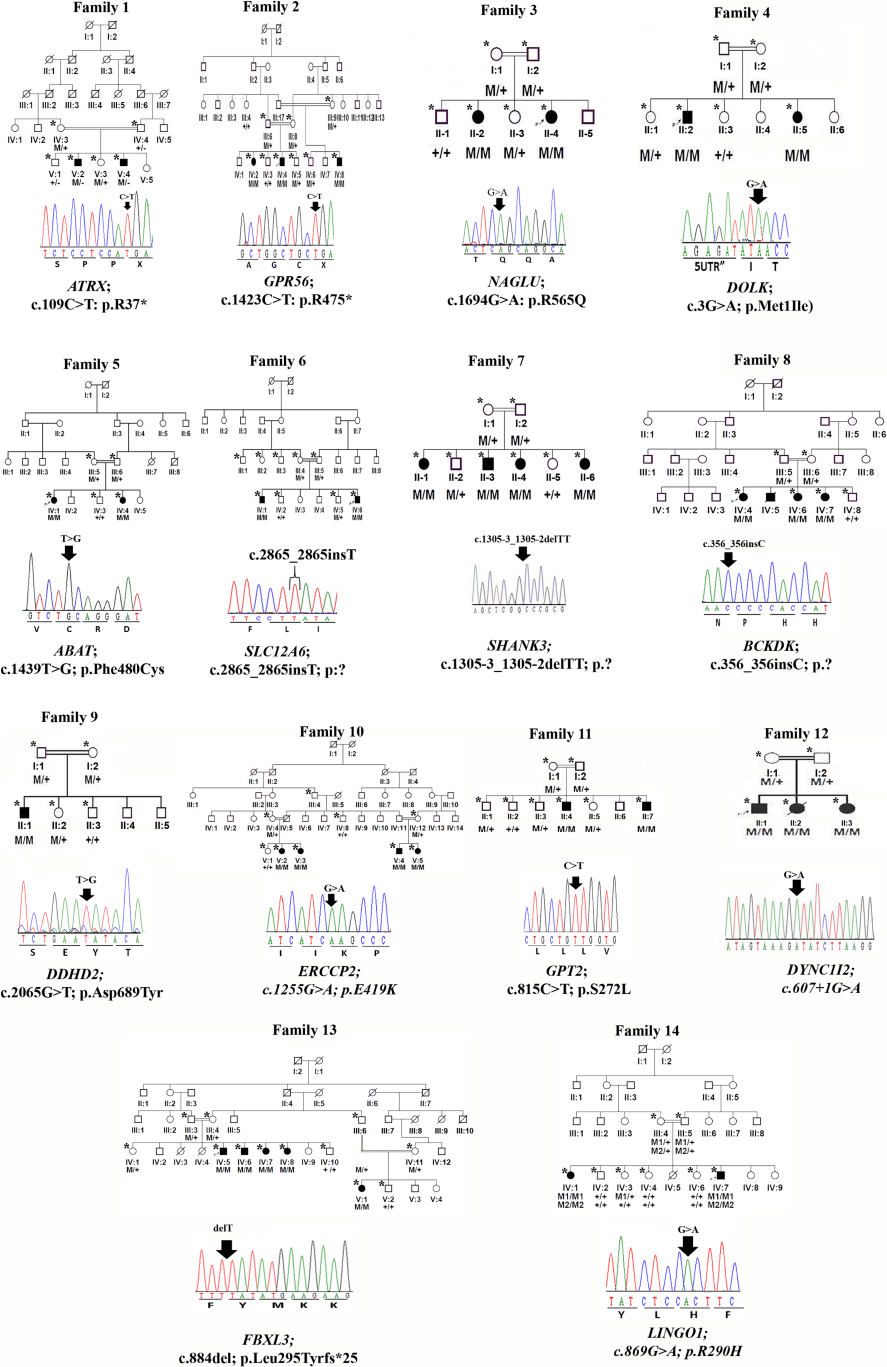
Family pedigrees and Sanger sequencing chromatogram and mutation positions of 14 families.

**Figure 2:**
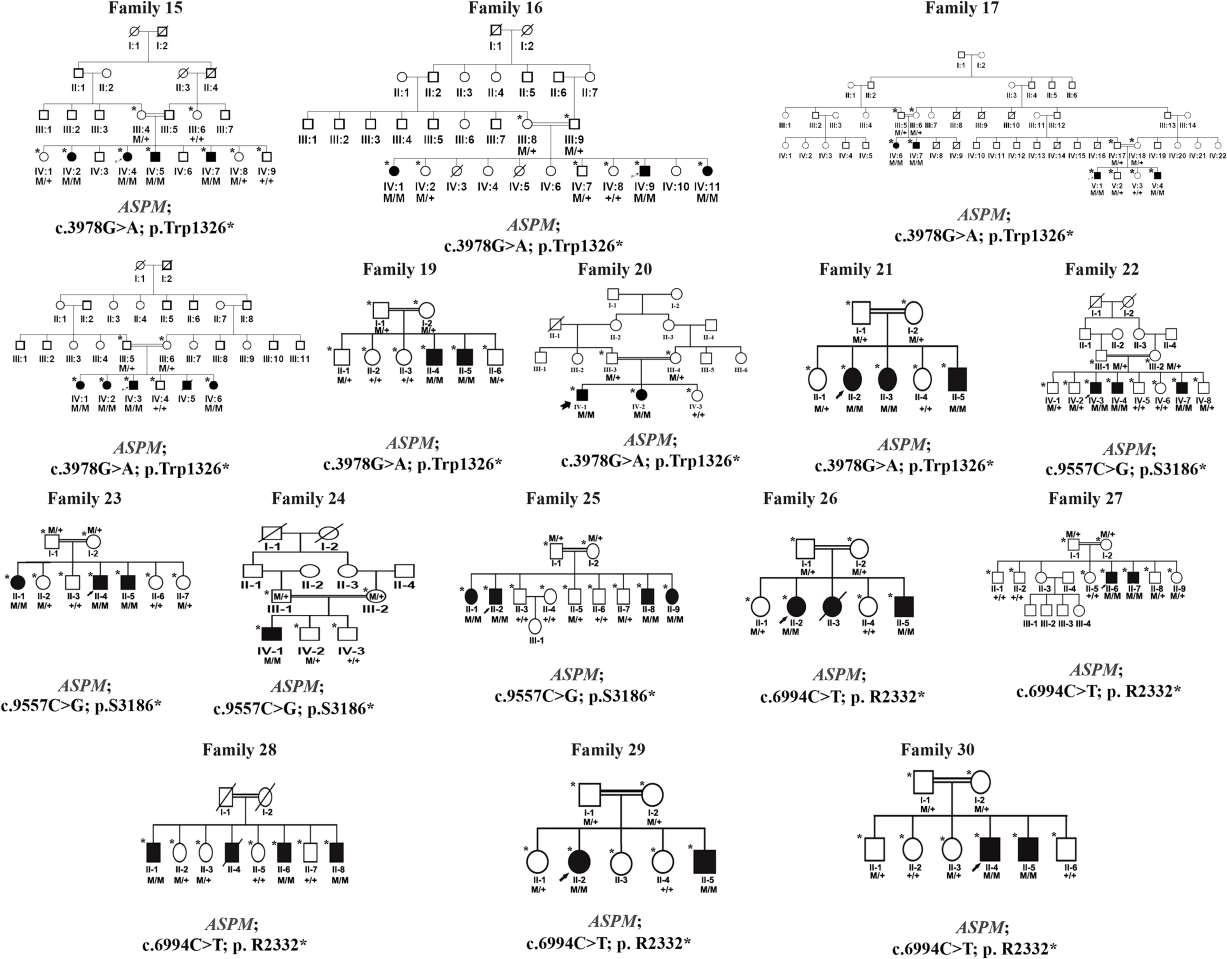
Families pedigrees of 16 families (family 15–family 30). All the families revealed autosomal recessive inheritance.

Peripheral blood was obtained in EDTA vacutainers from all available individuals in each family, as indicated by asterisks in the pedigrees shown in Figures 1, 2. DNA was extracted and quantified using standard protocols (14, 15).

### 2.3 WES and data analysis

In families (F1-F18), whole exome sequencing was performed on a single affected individual from each family using SureSelect Human All Exon v6 reagents (Agilent Technologies). The Illumina HiSeq4000 sequencer was used for sequencing with 150 bp paired-end reads, achieving an average of 100X coverage per targeted base. The exome data were analyzed using an in-house customized pipeline incorporating published algorithms, including the Burrows-Wheeler aligner tool (BWA) (16), SAMtools (17), PICARD, and the Genome Analysis Toolkit (GATK) (18). Sequenced reads were aligned to the GRCh37/hg19 (19) reference human genome, and the filtering of variants was performed as described in previous studies (14, 20).

To identify the causative variant in families F19-F30, whole exome sequencing was performed using similar reagents and platforms as those used for families F1-18, but with 100-bp paired-end reads yielding an average of 60x coverage per targeted base using the ligation-mediated di-base detection system as previously described (15, 21). All reads obtained were aligned to the human assembly hg19 (GRCh37) using Burrows-Wheeler Aligner (BWA v. 0.7.5). Variant calling was performed using the Pindel, SAM tools, and ExomeDepth algorithm. Subsequently, the final variant calling format file (VCF) was generated, uploaded, and analyzed using the Illumina Base Space online tool (22, 23). Pathogenic, likely pathogenic, and variants of uncertain significance were identified according to the American College of Medical Genetics and Genomics (ACMG) guidelines (24).

### 2.4 Variant classifications and prioritization

Variants were classified according to the ACMG guidelines and filtered using standard methods (20, 25). In short, after the initial filtration, the remaining variants were evaluated based on conservation scores (by GERP++) (26) and NCBI homologene (http://www.ncbi.nlm.gov/homologene/).

The pathogenicity index was assessed using MutationTaster, Polymorphism Phenotyping V2 (PolyPhen-2), and Sorting Intolerant From Tolerant (SIFT). The frequency of each variant in the general population was determined using databases such as ExAC, gnomAD, the 1,000 Genomes Project, local controls, and the professional version of the Human Gene Mutation Database (23). The analysis was conducted using Illumina GenomeStudio software (http://www.illumina.com/software/genomestudio_software.ilmn). PROVEAN and EditSeq were used to analyze frameshift variants (27).

### 2.5 Protein 3D modeling for novel mutations

The amino acid sequence of 4-aminobutyrate aminotransferase (ABAT), Phospholipase DDHD2, Assembly factor for spindle microtubules (ASPM), and DNA repair factor IIH helicase subunit XPD (ERCC2) encoding proteins were retrieved from the UniProt database using their respective accession numbers. The 3D structures of the mutated protein were generated using MODELLER (9.19), which assists in 3D structure prediction by satisfying spatial restraints based on the evaluation score. PyMOL was used to visualize interatomic interactions (28, 29).

RAMPAGE and ERRAT were used for model evaluation and to assess the distribution of residues in the preferred, permitted, and outlier areas. RAMPAGE creates a Ramachandran plot for model evaluation, while ERRAT provides overall model confidence and quality.

### 2.6 Sanger sequencing

The variants identified after WES data analysis were confirmed through Sanger sequencing in all available family members, as described previously (29, 30). Primers for all identified variants were designed using Primer 3 and/or Exon Primer and are available upon request.

## 3 Results

### 3.1 Recruitment of families

In the present study, we examined 30 unrelated consanguineous families with affected individuals exhibiting hallmark features of NDDs from the Punjab and KPK provinces of Pakistan, with likely autosomal recessive inheritance patterns. The affected individuals underwent clinical and genetic evaluations. Detailed clinical examinations were conducted in local hospitals. Molecular analyses were conducted at the University of Geneva School of Medicine, Department of Genetic Medicine and Development, Geneva, Switzerland (F1-F18) and the Medical Genomics Research Department, King Abdullah International Medical Research Center (KAIMRC), King Saud Bin Abdul Aziz University for Health Sciences, Ministry of National Guard Health Affairs (MNGH), Riyadh, Saudi Arabia (F19-F30).

### 3.2 Clinical evaluation

In our study cohort of 30 families comprising 80 affected members, the mean age was observed to be 12 ± 2.5 years, with a male-to-female ratio of 1:1. Out of the 30 families, 10 families had more than 2 affected individuals. The affected individuals from each family showed various types of phenotypical representation, as highlighted in Tables 1, 2.

**Table 1:** Novel mutations, disease type, and clinical description of patients identified in the present study.

Family	Gene	Associated disorder	OMIM	NM_	Mutation/ ACMG classification	Total mutations reported	Novel/ reported	The phenotype observed in our cohort	Reported phenotype in literature OMIM
5	*ABAT* *(AR)*	*GABA-transaminase deficiency*	*613,163*	*NM_020686.6*	c.1439 T > G; p.Phe480Cys Variant of uncertain significance (VUS; Class 3)	17	Novel	The affected individuals presented with moderate ID, developmental delay, speech problems, and aggressive behavior.	Central Nervous System Hypotonia - Psychomotor retardation, severe - Seizures, refractory - Hyperreflexia - Tonic posturing - Lethargy - Leukodystrophy (in 1 of 2 patients) - Agenesis of the corpus callosum (in 1 of 2 patients) - Cerebellar hypoplasia (in 1 of 2 patients)
6	*SLC12A6* *(AR)*	*Agenesis of the corpus callosum with peripheral neuropathy*	*218,000*	*NM_133647.2*	c.2866dup; p.Ser956PhefsTer27 Pathogenic (Class 1)	29	Novel	The affected individuals presented with similar phenotypes and showed early onset severe ID, hypotonia, areflexia, and mild dysmorphic facial features. They were dysarthric, had strabismus, and were unable to stand or walk.	Central Nervous System - Delayed motor milestones - Developmental delay - Hypotonia, generalized - Mental retardation, mild to severe - Individuals can stand or walk with support by 4 to 6 years of age - Seizures - Agenesis of the corpus callosum - Enlarged ventricles - Axonal swelling of spinal nerve roots and cranial nerves Peripheral Nervous System - Peripheral motor neuropathy, severe - Peripheral sensory neuropathy, severe - Areflexia
7	*SHANK3* *(AR)*	*Phelan-McDermid syndrome*	*606,232*	*NM_001372044.2*	c.1305-3_1,305-2delTT; p.? VUS; Class 3	377	Novel	They presented with severe ID, severely delayed developmental milestones, hypotonia, severely impaired speech, aggressive behavior, and mild facial dysmorphism.	Central Nervous System - Global developmental delay - Delayed motor development - Absent or delayed speech development - Compromised expressive language development, severe - Mental retardation, moderate to severe - Generalized hypotonia - Seizures
									Peripheral Nervous System - Increased tolerance to pain - Hyporeflexia, neonatal - Abnormal reflexes Behavioral abnormalities
8	*BCKDK* *(AR)*	*Branched-chain keto acid dehydrogenase kinase deficiency*	*614,923*	*NM_005881.4*	c.360dupC; p.Thr121HisfsTer7 Likely Pathogenic (Class 2)	08	Novel	The affected sisters presented with the severe phenotype of the neurobehavioral disorder, including ID, microcephaly, and behavioral abnormalities.	autism, epilepsy, intellectual disability, and reduced branched-chain amino acids (BCAAs), EEG showed left temporal epileptogenic dysfunction
9	*DDHD2* *(AR)*	*Spastic paraplegia 54, autosomal recessive*	*615,033*	*NM_015214.3*	c.2065G > T; p.Asp689Tyr VUS; Class 3	33	Novel	The affected individuals showed delayed psychomotor and intellectual development and early onset rigidity of lower limbs due to hypertonia.	Central Nervous System - Delayed psychomotor development - Mental retardation - Spastic paraplegia - Hyperreflexia - Extensor plantar responses - Lower limb weakness - Dysarthria - Thin corpus callosum - Periventricular white matter abnormalities - Abnormal lipid peak on brain MRS
10	*ERCC2* *(AR)*	*Trichothiodystrophy 1, photosensitive*	*601,675*	*NM_000400.4*	c.1255G > A; p.Glu419Lys VUS; Class 3	141	Novel	The affected individuals showed severe ID, developmental delay, delayed speech to absent speech, microcephaly (<1 centile), mild hypotonia, and mild facial dysmorphism, such as a relatively large nose with a beaked tip.	Central Nervous System - Mental retardation (IQ 45–58) - Poor gross and fine motor coordination - Speech delay - Diffuse signal hyperintensity of white matter seen on MRI thin hair - sparse hair, ichthyosis, cataracts and short stature

**Table 2:** Reported mutations, disease type, and clinical description of patients identified in the present study.

Family	Gene	Associated disorder	OMIM	NM_	Mutation	Total mutations reported	Novel/ Reported	The phenotypes observed in our cohort	Reported phenotype in literature OMIM
1	*ATRX* *(AR)*	Intellectual disability-hypotonic facies syndrome, X-linked	309,580	NM_000489.6	c.109C > T; p.Arg37* Pathogenic (Class 1)	210	Reported	The affected individuals manifested moderate to severe ID, aggressive behavior, language problems, mild microcephaly, and mild facial dysmorphic features.	Short stature, microcephaly, hypogonadism - Small testes - Hypoplastic scrotum, kyphoscoliosis, brachydactyly, mental retardation, severe, profound - Hypotonia, early - Hypertonia of lower limbs, later - Hyperreflexia - Seizures (in 35%)
2	*ADGRG1 (GPR56)* *(AR)*	*Polymicrogyria, bilateral frontoparietal*	*606,854*	*NM_005682.7*	c.1423C > T; p.Arg476* Pathogenic (Class 1)	45	Reported	The affected individuals showed severe ID manifesting with global motor and developmental delay, ataxic gait, absent or delayed language, hypotonia, and aggressive behavior.	Neurological features reported: developmental delay - Mental retardation, moderate to severe - Increased muscle tone - Hyperreflexia - Seizures - Truncal ataxia - Finger dysmetria - Brainstem hypoplasia - Cerebellar hypoplasia Eyes abnormalities: - Dysconjugate gaze - Esotropia - Nystagmus - Strabismus - Exotropia
3	*NAGLU* *(AR)*	*Mucopolysaccharidosis type IIIB (Sanfilippo B)*	*252,920*	*NM_000263.4*	***c.1694G > A;*** ***p.Arg565Gln*** ***Pathogenic (Class 1)***	265	Reported	The affected sisters showed severe phenotypes, including progressive neurological deterioration, developmental delays (speech delay was more than motor delay), mild hearing loss, severe ID, and coarse facial features.	Central Nervous System - Neurologic deterioration, progressive - Slowing mental development by 1.5 to 3 years of age - Sleep disturbances - Mental retardation - Seizures, Hyperactivity - Aggressive behavior, hearing loss
4	*DOLK* *(AR)*	Congenital disorder of glycosylation, type Im	610,768	NM_014908.4	c.3G > A; p.Met1Ile Pathogenic (Class 1)	22	Reported	The affected individual revealed delayed developmental milestones, psychomotor development, and speech.	Dilated cardiomyopathy, ichthyosis, central nervous system - Hypotonia, profound muscular (in some patients) - Seizures (in some patients) - Hypsarrhythmia (in some patients), sparse eyebrows - Sparse eyelashes
11	*GPT2* *(AR)*	*Neurodevelopmental disorder with microcephaly and spastic paraplegia*	*616,281*	*NM_133443.4*	c.815C > T; p.Pro272Leu **Pathogenic (Class 1)**	19	Reported	The affected individuals exhibited severe ID, delayed developmental milestones with motor delay, speech problems, postnatal microcephaly, hypotonia, aggressive behavior, and spastic paraplegia in one patient.	Central Nervous System - Microcephaly Global developmental delay - Impaired intellectual development, moderate to severe - Delayed speech - Absent speech - Dysarthria - Seizures (in some patients) - Axial hypotonia - Walking difficulties
12	*DYNC1I2* *(AR)*	*Neurodevelopmental disorder with microcephaly and structural brain anomalies*	*618,492*	*NM_001378.3*	*c.607 + 1G > A;* *p.?* Pathogenic (Class 1)	04	Reported	The family comprised three affected individuals who exhibited mild to moderate ID, microcephaly, short stature, developmental delay, aggressive behavior, and mild facial dysmorphism. These facial features include upslanting palpebral features, anteverted nares, and a bulbous nose.	Central Nervous System - Microcephaly, Short stature, Global developmental delay, severe - Impaired intellectual development - Speech delay - Delayed walking - Inability to walk - Spasticity - Hyperreflexia - Seizures (in some patients),
13	*FBXL3* *(AR)*	*Intellectual developmental disorder with short stature, facial anomalies, and speech defects*	*606,220*	*NM_012158.4*	c.884delT; p.(Leu295Tyrfs*25) **Pathogenic (Class 1)**	04	Reported	All the affected patients had similar clinical presentations of ID, short stature, and mild facial dysmorphology.	Central Nervous System - Global developmental delay - short stature - Impaired intellectual development, variable - Delayed speech - Poor speech - Dysarthria - Delayed walking
14	*LINGO1* *(AR)*	*Intellectual developmental disorder, autosomal recessive 64*	*618,103*	*NM_032808.7*	c.869G > A; p.Arg290His Pathogenic (Class 1)	05	Reported	Affected siblings have similar severe phenotypes of neurobehavioral disorder, including severe ID, motor and speech delay, microcephaly, and behavioral abnormalities.	Central Nervous System Microcephaly, - Delayed psychomotor development - Intellectual disability, moderate to severe - Poor or absent speech - Spasticity (in some patients) - Hypertonia (in some patients) - Seizures (in 1 patient)
15	*ASPM* *(AR)*	Microcephaly 5, primary, autosomal recessive	608,716	NM_018136.5 1	c.3978G > A; Trp1326* Pathogenic (Class 1)	264	Reported	Clinical analysis of the affected individual revealed variable head circumference, cognitive impairment, aggressive behaviors, and other related behavioral problems. Delayed language development; HC revealed a microcephalic diagnosis.	Central Nervous System - Mental retardation, mild to severe - Delayed motor development - Delayed language development - Seizures (less common) - Hypoplastic corpus callosum - Small cerebral cortex - Simplified cortical gyration pattern - Small pons - Small frontal lobe
16	*ASPM* *(AR)*	Microcephaly 5, primary, autosomal recessive	608,716	2	c.3978G > A; Trp1326* Pathogenic (Class 1)		Reported		Truncating mutations resulted in a severe phenotype with childhood-onset, whereas missense mutations resulted in a mild phenotype with a relatively late age at onset
17	*ASPM* *(AR)*	Microcephaly 5, primary, autosomal recessive	608,716	3	c.3978G > A; Trp1326* Pathogenic (Class 1)		Reported		
18	*ASPM* *(AR)*	Microcephaly 5, primary, autosomal recessive	608,716	4	c.3978G > A; Trp1326* Pathogenic (Class 1)		Reported		
19	*ASPM* *(AR)*	Microcephaly 5, primary, autosomal recessive	608,716	5	c.3978G > A; Trp1326* Pathogenic (Class 1)		Reported		
20	*ASPM* *(AR)*	Microcephaly 5, primary, autosomal recessive	608,716	6	c.3978G > A; Trp1326* Pathogenic (Class 1)		Reported		
21	*ASPM* *(AR)*	Microcephaly 5, primary, autosomal recessive	608,716	7	c.3978G > A; Trp1326* Pathogenic (Class 1)		Reported		
22	*ASPM* *(AR)*	Microcephaly 5, primary, autosomal recessive	608,716	1	c.9557C > G; p.S3186* Pathogenic (Class 1)		Reported		
23	*ASPM* *(AR)*	Microcephaly 5, primary, autosomal recessive	608,716	2	c.9557C > G; p.S3186* Pathogenic (Class 1)		Reported		
24	*ASPM* *(AR)*	Microcephaly 5, primary, autosomal recessive	608,716	3	c.9557C > G; p.S3186* Pathogenic (Class 1)		Reported		
25	*ASPM* *(AR)*	Microcephaly 5, primary, autosomal recessive	608,716	4	c.9557C > G; p.S3186* Pathogenic (Class 1)		Reported		
26	*ASPM* *(AR)*	Microcephaly 5, primary, autosomal recessive	608,716	1	c.6994C > T; p. R2332* Pathogenic (Class 1)		Reported		
27	*ASPM* *(AR)*	Microcephaly 5, primary, autosomal recessive	608,716	2	c.6994C > T; p. R2332* Pathogenic (Class 1)		Reported		
28	*ASPM* *(AR)*	Microcephaly 5, primary, autosomal recessive	608,716	3	c.6994C > T; p. R2332* Pathogenic (Class 1)		Reported	
29	*ASPM* *(AR)*	Microcephaly 5, primary, autosomal recessive	608,716	4	c.6994C > T; p. R2332* Pathogenic (Class 1)		Reported	
30	*ASPM* *(AR)*	Microcephaly 5, primary, autosomal recessive	608,716	5	c.6994C > T; p. R2332* Pathogenic (Class 1)		Reported	

### 3.3 Genetic analysis

Exome sequencing revealed 18 likely causative homozygous variants in the 30 families: 6 novel and 12 reported in 16 different genes. These variants were segregated with the disease phenotype in all 30 families. Variants in the *ASPM* gene were the cohort’s most observed mutations, present in 16 out of 30 families (53.3%). Furthermore, nonsense variants were found in 18 families (18/30: 60%).

A comparison of the phenotypes of 30 variants in genes that cause NDDs showed phenotypic overlap with previously reported cases (Tables 1, 2). Some deviations were observed, which may result from the variable effects of allelic mutations or the influence of genetic modifier variants, which may be particularly relevant in populations with a high consanguinity rate. Additionally, other segregating pathogenic variants in other genes may also be responsible for unusual clinical manifestations in some affected individuals.

### 3.4 Known pathogenic mutations in known autosomal recessive genes

We identified 24 already reported/published variants in 9 different genes. These included three nonsense mutations: *ATRX* (c.109C > T; p.Arg37*), *GPR56* (c.1423C > T; p.Arg475*), and *DOLK* (c.3G > A; p.Met1Ile). Additionally, there were three nonsense variants in the *ASPM* gene: c.3978G > A (Trp1326*) in seven families, c.6994C > T (p. R2332*) in five families, and c.9557C > G (p.S3186*) in four families. All families with *ASPM* nonsense variants were not directly related and were recruited from different cities in the KPK province. The variant c.3978G > A (Trp1326*) is known as a founder mutation in the KPK province. Interviews with the parents of other patients confirmed that they were unrelated. Three missense mutations were identified: *NAGLU* (c.1694G > A; p.Arg565Gln), *GPT2* (c.815C > T; p.Ser272Leu), and *LINGO1* (c.869 G > A; p.Arg290His). Additionally, a one-base pair in-frame deletion was found in *FBXL3* (c.885delT; p.Leu295Phefs25*), and a splice site variant was found in *DYNC1I2* (c.607 + 1G > A) (Tables 1, 2; Figures 1, 2). Sanger sequencing confirmed the segregation of the identified variants with the disease phenotype within the families compatible with an autosomal recessive mode of inheritance (Figures 1, 2).

### 3.5 Novel likely pathogenic mutations in known autosomal recessive genes

We identified six novel mutations in six different genes associated with NDDs:

- **ABAT Gene** (NM_020686.6): A homozygous missense mutation (c.1439 T > G; p.Phe480Cys) associated with GABA-transaminase deficiency (OMIM 613163).
- **SLC12A6 Gene** (NM_133647.2): A homozygous one-base pair frameshift insertion mutation (c.2865_2865insT; p.Glu955Asnfs*5) associated with agenesis of the corpus callosum with peripheral neuropathy (OMIM 218000).
- **SHANK3 Gene** (NM_001372044.2): A splice site variant (c.13zc05-3_1,305-2delTT; p.Gln29-_Gly305del) associated with Phelan-McDermid syndrome (OMIM 606232).
- **BCKDK Gene** (NM_005881.4): A homozygous one-base pair frameshift insertion mutation (c.356_356insC; p.Thr121HisfsTer7) that causes branched-chain ketoacid dehydrogenase kinase deficiency (BCKDK) (OMIM 614923).
- **DDHD2 Gene** (NM_015214.3): A homozygous missense variant (c.2065G > T; p.Asp689Tyr) responsible for spastic paraplegia 54, autosomal recessive (OMIM 615033).
- **ERCC2 Gene** (NM_000400.4): A homozygous missense variant (c.1255G > A; p.Glu419Lys) that underlies trichothiodystrophy 1, photosensitive (OMIM 601675) (Tables 1, 2).

**Figure 3:**
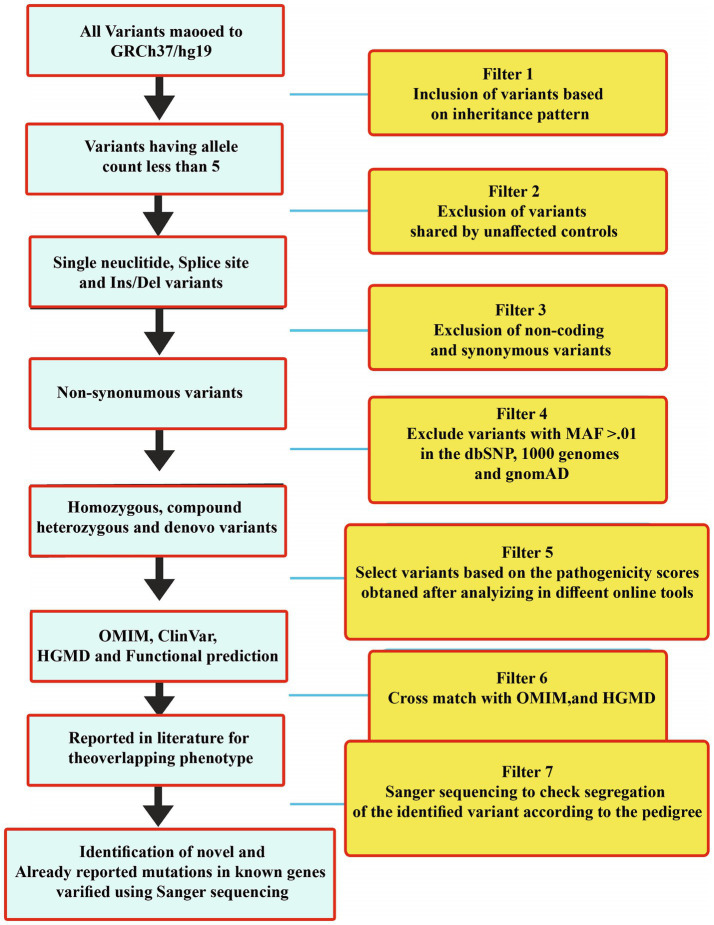
WES filtration steps showing details of each step that was followed to identify variants in all the 30 families. Variants were classified according to the ACMG classification.

The WES and filtration steps are shown in a flow sheet in Figure 3. Variants were classified according to the ACMG guidelines into five categories: pathogenic (class-1), likely pathogenic (class-2), variant of uncertain significance (class-3), likely benign (class-4), and benign (class-5) (Table 2).

The identified mutations were not observed in gnomAD, ExAC, the 1,000 Genomes Project, or in-house exomes. The sequence variations/mutations detected in the present study were characterized using the Human Genome Variation Society (HGVS, http://www.hgvs.org/mutnomen/) nomenclature. Identified variants were classified as either novel or already reported variants using online available databases such as PubMed (https://www.ncbi.nlm.nih.gov/pubmed), ClinVar (https://www.ncbi.nlm.nih.gov/clinvar/), and HGMD (http://www.hgmd.cf.ac.uk/ac/validate.php).

### 3.6 Molecular modeling analysis

**Figure 4:**
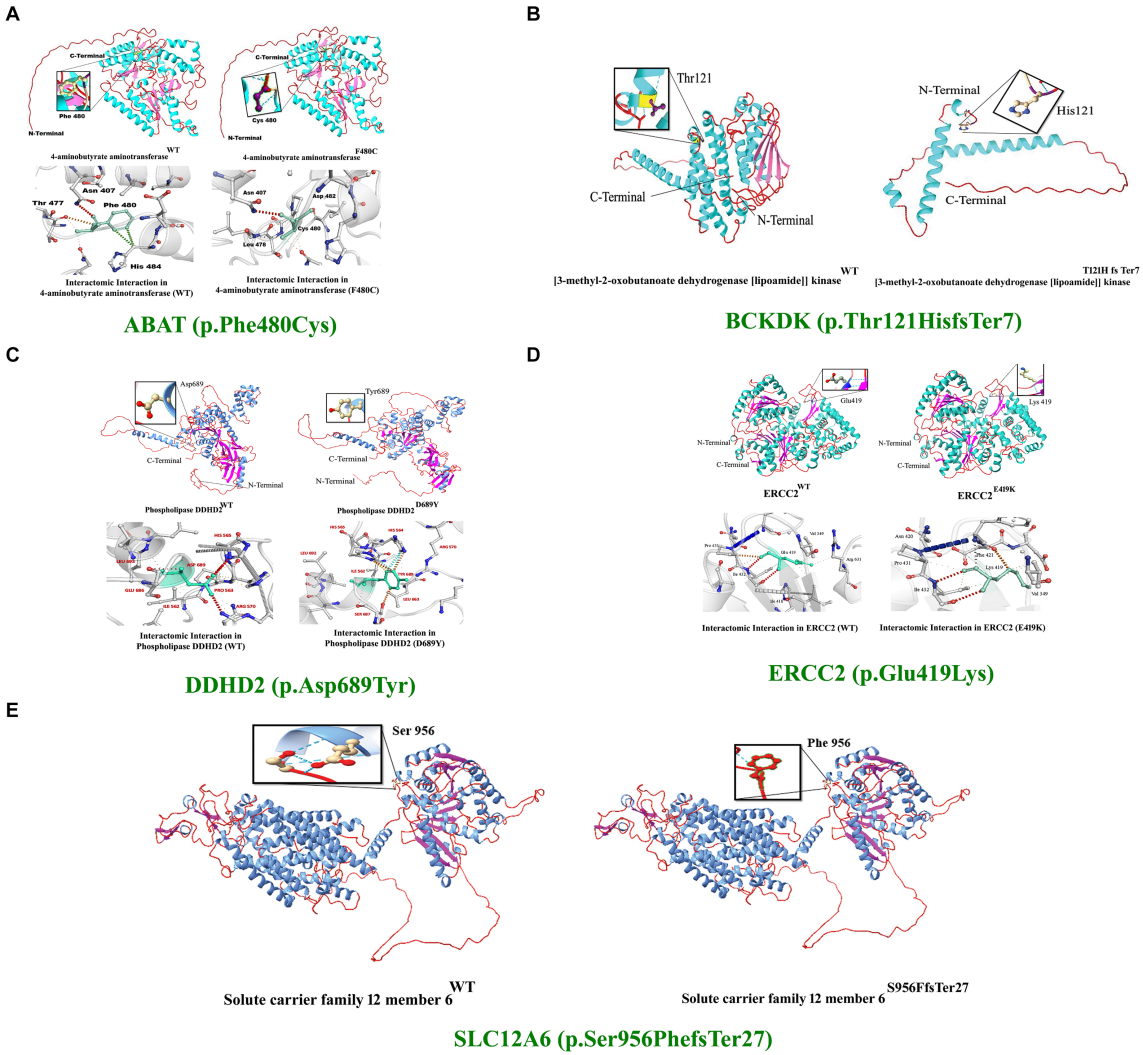
3D protein modeling for the variant identified in five proteins, including ABAT, BCKDK, DDHD2, ERCC2, and SLC12A6. Protein modeling revealed that the identified variants in these proteins resulted in substantial changes in the secondary structures that might lead to improper folding, structure, and function, causing severe NDDs in the affected individuals of the families.

*In silico* homology modeling was conducted for both wild-type and mutant forms of ABAT, DDHD2, and ERCC2 proteins. Additionally, three-dimensional protein models for wild-type and mutant forms of *ABAT* (p.Phe480Cys), *DDHD2* (p.Asp689Tyr), and *ERCC2* (p.Glu419Lys) were predicted and evaluated using standard methods. The Ramachandran plot indicated 94 and 99% of residues in the wild-type and mutant structures, respectively, lie in allowed regions of torsion angles. The 3-D structures were then subjected to the ERRAT protein structure verification server, which provided the overall quality scores of 89 and 99% for the predicted protein models (Figures 4A–E).

The 4-aminobutyrate aminotransferase enzyme helps break down a brain chemical (neurotransmitter) called GABA when it is not needed. GABA normally helps slow down (inhibit) brain cell activity when necessary to prevent the brain from being overloaded with too many signals. This enzyme is found in the matrix of mitochondria (31). Substitution of phenylalanine (Phe), which is a hydrophobic, very large (189.9 Å), aromatic, and non-polar amino acid, with cysteine, which is also hydrophobic but small (108.5 Å) and non-polar, was observed. However, using DUET, ENCoM, SDM, and mCSM, we predicted that the Phe480Cys mutation would cause changes in the ΔΔG of −2.65, −0.640, −1.39, and − 2.426 kcal/mole, respectively (Figure 4A).

The Excision Repair Cross-Complementation Group 2 (ERCC2) gene, also known as XPD, encodes a DNA helicase essential for the nucleotide excision repair (NER) pathway, which is crucial for maintaining genomic stability by repairing DNA damage caused by ultraviolet (UV) light and other environmental mutagens. ERCC2 plays a role in transcription-coupled repair (TCR), a sub-pathway of NER that specifically targets and repairs DNA lesions that block transcriptions. As part of the transcription factor IIH (TFIIH) complex, ERCC2 is also involved in the initiation of transcription by RNA polymerase II, highlighting its significance in regulating gene expression and maintaining genome integrity (32). The *ERCC2* mutation reported in our study is predicted to change the stability of the deduced protein due to deranged physiochemical properties resulting from the amino acid substitution (p.Glu419Lys). Amino acid point mutations may change protein structure and function. However, using DUET, ENCoM, SDM, and mCSM, we predicted that the Glu419Lys mutation would cause changes in the ΔΔG of −1.774, 0. 156, −0.63, and − 1.917 kcal/mole, respectively (Figure 4B).

The *DDHD2* gene encodes a phospholipase enzyme known as phospholipase A1 (PLA1), which plays a crucial role in lipid metabolism by hydrolyzing phospholipids and releasing fatty acids. This enzyme is essential for maintaining the balance of lipid species within cellular membranes, thereby contributing to lipid homeostasis and membrane fluidity. DDHD2 is involved in vesicle trafficking and membrane remodeling, processes that are vital for intracellular transport and membrane dynamics (33). In DDHD2, the substitution of aspartic acid (Asp), a small (111.1 Å), hydrophilic, acidic, and polar amino acid, with tyrosine (Tyr), a very large (193.6 Å), neutral, aromatic, and uncharged polar amino acid, can significantly alter the protein’s structure and function..

Amino acid point mutations may change the protein structure and function. However, no method directly predicts the impact of these mutations on the structure. However, using DUET, ENCoM, SDM, and mCSM, we predicted that the Asp 689Tyr mutation would cause changes in the ΔΔG of −1.387, 0. 398, −0.97, and − 1.236 kcal/mol, respectively. These changes indicate that the mutation would greatly destabilize the protein structure and disrupt its function. (Figures 4C–E).

The branched-chain ketoacid dehydrogenase kinase [*BCKDK* (*SCE223*)] gene encodes an enzyme that plays a pivotal role in the regulation of branched-chain amino acid (BCAA) metabolism.

BCKDK phosphorylates and inhibits the branched-chain alpha-keto acid dehydrogenase complex (BCKD), which is responsible for the catabolism of BCAAs such as leucine, isoleucine, and valine. By inhibiting BCKD, BCKDK helps maintain the balance of BCAAs in the body, preventing their excessive breakdown and ensuring their availability for protein synthesis and other vital functions. Proper BCAA regulation is crucial for normal growth, energy production, and the function of various organs, including the brain (34).

The BCKDK (p.Thr121Hisfs*7) and SLC12A6 (p.Ser956Phefs*27) mutations reported in our study are predicted to change the respected amino acids and result in frameshifts and premature stop codons. Thus, a substantial part of the functional protein will be lost as both mutations are located in the critical domains of the protein. These mutations might lead to the loss of protein functions, causing the proteins to possibly be degraded.

The *ASPM* gene encodes a protein that plays a critical role in neurogenesis, particularly in the regulation of mitotic spindle function during cell division in neural progenitor cells. ASPM is essential for maintaining the symmetric division of these progenitor cells, ensuring the proper generation and proliferation of neurons in the developing brain. This protein localizes to the centrosome and the spindle poles, where it regulates spindle orientation and helps ensure accurate chromosome segregation (35).

**Figure 5:**
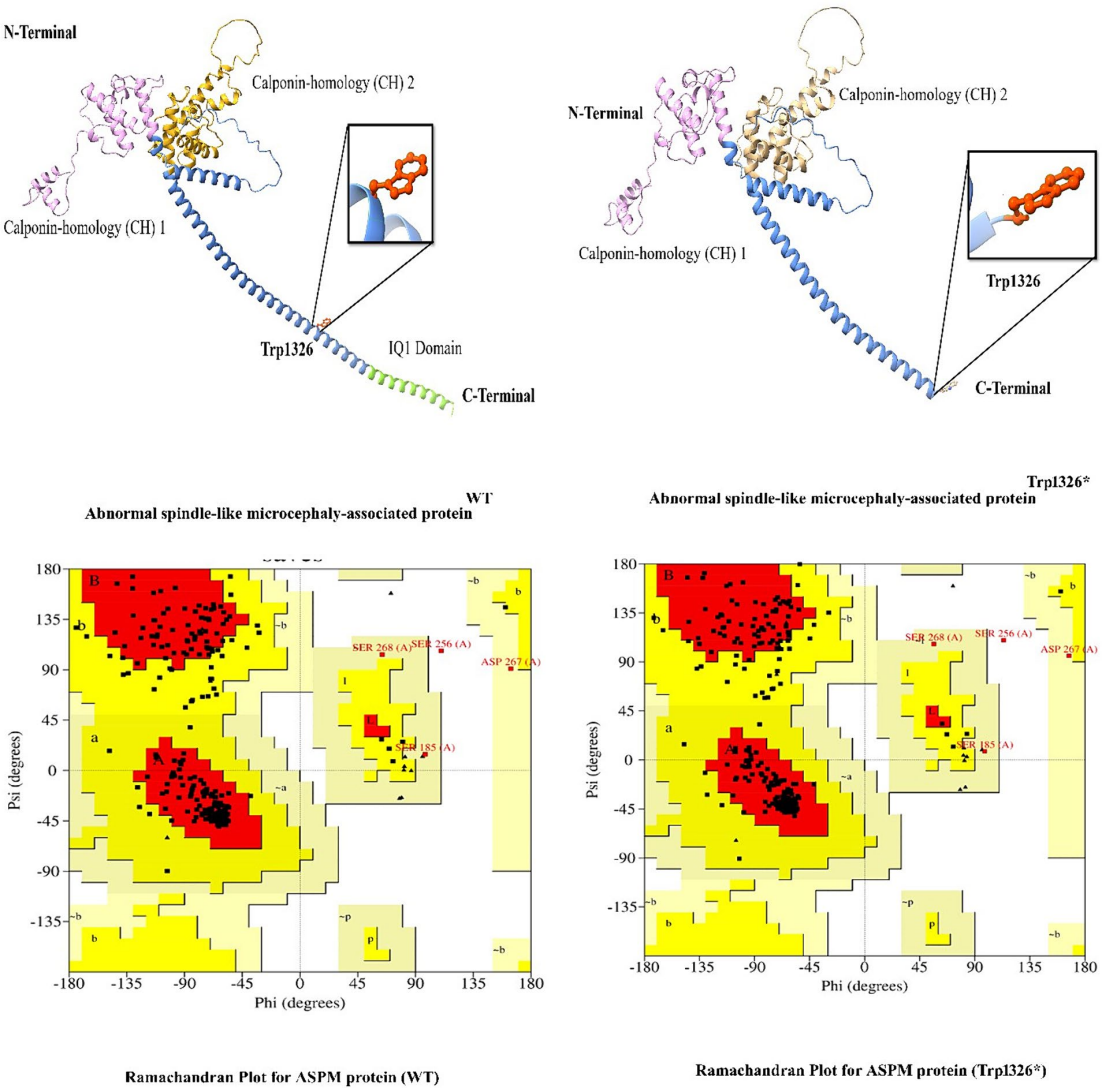
3D protein modeling for the hotspot mutation (Trp1326*) identified in the present study in the *ASPM* gene.

In this study, we employed *in silico* methodologies such as homology modeling for both wild-type and mutant proteins. ASPM is a 3,477 amino acid-long protein. To observe the effect of the identified mutation (Trp1326*) on protein structure, we analyzed the sequence between the Calponin-homology (CH) 1 domain and the IQ1 domain since the mutation lies between the Calponin-homology (CH) 2 and IQ1 domains of the ASPM protein. A nonsense mutation in ASPM led to a premature termination of the ASPM protein at amino acid position 1,326. This mutation is located immediately before the IQ domains of the ASPM protein, resulting in a premature protein that lacks all IQ domains. The most commonly reported mutation (Trp1326*) in ASPM from our study is predicted to change the secondary structure of the ASPM protein. As this nonsense mutation is located in the first half of the protein (total ASPM protein is 3,477 amino acids), it will likely affect the stability and function of the deduced protein (Figure 5).

## 4 Discussion

NDDs are a heterogeneous group of disorders associated with intellectual disability (ID), global developmental delay (GDD), epilepsy, mild-to-severe microcephaly, autism spectrum disorders (ASD), attention-deficit/hyperactivity disorders (ADHD), and learning disorders. These disorders lack precise boundaries in their clinical definitions, epidemiology, genetics, and other associated phenotypes, which can result in significant limitations in intellectual functioning and adaptive behavior (8). Additional features such as hearing impairment, speech and language disorders, ID, epilepsy, and learning disorders are commonly observed in NDD patients (4).

Approximately 40% of NDD ID conditions remain molecularly undiagnosed, and approximately 50% have an environmental etiology (36) Environmental factors include improper care during pregnancies, multiple pregnancies with short intervals, poor nutrition, brain ischemia (prenatal/perinatal), inadequate medical services, and postnatal infections. The ID has a genetic etiology, such as mutations in genes and rare chromosomal abnormalities, which substantially impact families and society.

We report the results of a study on NDDs in a cohort of 30 families from the Pakistani population. Disease-causing variants in known NDD genes were identified in all the reported 30 families. The investigated families exhibited mutations in 15 known genes, resulting in diverse clinical phenotypes that are presented in Tables 1, 2. These mutations include six novel and 24 previously reported mutations..

The 24 reported mutations included the following:

- In **family 1**, two affected individuals were found to have a nonsense mutation (c.109C > T; p.Arg37*) in *ATRX*, which is associated with intellectual disability- In hypotonic facies syndrome (OMIM 309580).
- In **family 2**, three affected individuals were found to have a nonsense mutation (c.1423C > T; p.Arg475*) in *GPR56*, which is associated with bilateral frontoparietal polymicrogyria (OMIM 606854).
- In **family 3**, two affected individuals were found to have a missense mutation (c.1694G > A; p.Arg565Gln) in *NAGLU*, which is associated with Mucopolysaccharidosis type IIIB (Sanfilippo B) (OMIM 252920).
- In **family 4**, two affected individuals were found to have a missense mutation (c.3G > A; p.Met1Ile) in DOLK, which is associated with a congenital disorder of glycosylation, type IM (OMIM 610768).
- In **family 11**, two affected individuals were found to have a missense mutation (c.815C > T; p.Ser272Leu) in GPT2, which is associated with neurodevelopmental disorder with microcephaly and spastic paraplegia (OMIM 616281).
- In **family 12**, two affected individuals were found to have a splice donor site mutation (c.607 + 1G > A) in DYNC1I2, which is associated with neurodevelopmental disorder with microcephaly and structural brain anomalies (OMIM 618492).
- In **family 13**, five affected individuals were found to have a frameshift deletion mutation (c.885delT; p.Leu295Phefs25*) in FBXL3, which is associated with neurodevelopmental disorder with intellectual disability, short stature, facial anomalies, and speech defects (OMIM 606220).
- In **family 14**, two affected individuals were found to have a missense mutation (c.869G > A; p.Arg290His) in LINGO, which is associated with intellectual developmental disorder, autosomal recessive 64 (OMIM 618103).

Families 15–30 were found to have mutations in the *ASPM* gene, which were associated with primary microcephaly (OMIM 608716), distributed as follows:

- Families 15–21: c.3978G > A (Trp1326*)
- Families 22–25: c.9557C > G (p.S3186*)
- Families 26–30: c.6994C > T (p.R2332*)

Similarly, we identified six novel mutations in the present investigation (Figure 2).

- In f**amily 5**, two affected individuals were found to have a homozygous missense mutation (c.1439 T > G; p.Phe480Cys) in the *ABAT* gene (NM_020686.6), which is associated with GABA-transaminase deficiency (OMIM 613163). To date, only 17 mutations have been identified in the *ABAT* gene (HGMD® Professional 2022.2).
- In f**amily 6**, two affected individuals were found to have a homozygous frameshift duplication mutation (c.2866dup; p.Ser956PhefsTer27) in the *SLC12A6* gene (NM_133647.2), which is associated with agenesis of the corpus callosum with peripheral neuropathy (OMIM 218000). To date, only 29 mutations have been identified in the *SLC12A6* gene (HGMD 2022.2).
- In **family 7**, four affected individuals were found to have a splice acceptor site deletion variant (c.1305-3_1,305-2delTT; p.Gln29_Gly305del) in the *SHANK3* gene (NM_001372044.2), which is associated with Phelan-McDermid syndrome (OMIM 606232). To date, 77 mutations have been identified in the *SHANK3* gene (HGMD 2022.2).
- In **family 8**, four affected individuals were found to have a homozygous duplication mutation (c.360dupC; p.Thr121HisfsTer7) in the *BCKDK* gene (NM_005881.4), which is associated with branched-chain ketoacid dehydrogenase kinase deficiency (OMIM 614923). To date, only eight mutations have been identified in the *BCKDK* gene (HGMD 2022.2).
- In **family 9**, a single affected individual was found to have a homozygous missense variant (c.2065G > T; p.Asp689Tyr) in the *DDHD2* gene (NM_015214.3), which is responsible for Spastic paraplegia 54, autosomal recessive (OMIM 615033). To date, only 33 mutations have been identified in the *DDHD2* gene (HGMD 2022.2).
- In **family 10**, four affected individuals were found to have a homozygous missense variant (c.1255G > A; p.Glu419Lys) in the *ERCC2* gene (NM_000400.4), which is responsible for underlying trichothiodystrophy 1, photosensitive (OMIM 601675). To date, only 141 mutations have been identified in the *ERCC2* gene (HGMD 2022.2).
- Mutations in the *ASPM* gene, causing primary microcephaly-5 (microcephaly and ID phenotypes), are the most common in our cohort (OMIM 608716). These results are not unexpected, as the prevalence of *ASPM* gene mutations is very high in the Pakistani population (31).

In the past few years, genetic, psychological, neuroanatomical, and molecular analyses of NDDs have led to the discovery of novel genes and the identification of associated pathways. These advances across all disciplines have brought us to a new scientific frontier that integrates molecular genetics with developmental cognitive neuroscience. Identifying variants in a population and discovering novel associated genes will enhance our current understanding of developmental brain disorders (37, 38). Furthermore, prenatal genetic screening is crucial to prevent the proliferation of severe NDDs and protect future generations (39–43). Preventing rare genetic disorders such as NDDs before they become common is imperative..

## 5 Conclusion

Our study highlights the clinical applicability of WES in elucidating the molecular basis of highly heterogeneous recessive disorders in populations such as Pakistan, where consanguineous marriages are common. Identifying variants in known genes, providing accurate molecular diagnoses, and pinpointing candidate genes in inbred families will facilitate the discovery of matching mutations in other populations. A significant strength of this study is the comprehensive presentation of clinical profiles alongside the reported candidate gene mutations. The extensive data collection in this manuscript is expected to enhance the interpretation of DNA variants in diagnostic exome studies for patients with difficult-to-diagnose neurodevelopmental disorders (NDDs). Future research using model systems may help unravel the functional integration of various molecular networks in normal brain development and activity, potentially leading to improved diagnosis and targeted therapies for neurological disorders..

Our study demonstrates the clinical applicability of WES in elucidating the molecular basis of highly heterogeneous recessive disorders in populations where consanguineous marriages are common, such as Pakistan. Identifying variants in the correctly reported genes, providing accurate molecular diagnoses, and identifying candidate genes for such conditions in consanguineous families will facilitate the identification of matching mutations in other populations. A significant strength of the present study is the comprehensive presentation of clinical profiles alongside the reported candidate gene mutations. The extensive data collection presented in this study is expected to enhance the interpretation of DNA variants in diagnostic exome studies for patients with difficult-to-diagnose NDDs. Future research using model systems may help unravel the functional integration of various molecular networks in normal brain development and activity, potentially leading to improved diagnosis of NDDs and a more effective targeted therapy.

## Data availability statement

The original contributions presented in the study are publicly available. This data can be found here: https://databases.lovd.nl/shared/references/DOI:10.3389/fmed.2024.1424753

## Ethics statement

The studies involving humans were approved by the University Hospitals of Geneva (Protocol CER 11–036) and the IRB of Khyber Medical University, Peshawar, Pakistan, and the Department of Life Sciences, School of Science, University of Management and Technology (UMT), Lahore, Pakistan. The studies were conducted in accordance with local legislation and institutional requirements. The participants’ legal guardians/next of kin provided written informed consent for participation in this study.

## Author contributions

SP: Methodology, Writing – original draft. SN: Writing – original draft, Methodology. MT: Methodology, Writing – original draft. AS: Methodology, Writing – review & editing. GZ: Data curation, Formal analysis, Software, Writing – review & editing. JA: Methodology, Writing – review & editing. FaS: Methodology, Writing – review & editing. SK: Methodology, Writing – review & editing. AJ: Data curation, Methodology, Writing – review & editing. NK: Methodology, Writing – review & editing. MK: Methodology, Writing – review & editing. QA: Methodology, Writing – review & editing. SaA: Methodology, Software, Writing – review & editing. SF: Methodology, Writing – review & editing. AW: Methodology, Writing – review & editing. AA: Methodology, Writing – review & editing. AH: Methodology, Writing – review & editing. FeS: Methodology, Software, Writing – review & editing. NU: Methodology, Writing – review & editing. BK: Writing – review & editing, Methodology. StA: Supervision, Writing – review & editing. KF: Supervision, Writing – review & editing. MU: Supervision, Writing – review & editing, Writing – original draft. MA: Supervision, Writing – review & editing.
